# Supplementary material for: Mutational scanning reveals oncogenic CTNNB1 mutations have diverse effects on signaling
Source: Nat Genet. 2026 Feb 2;58(2):366–75. doi: 10.1038/s41588-025-02496-5 (PMC12900645; doi:10.1038/s41588-025-02496-5)
Supplement: Supplementary file 1 — Supplemental Figs. 1–9. [file 41588_2025_2496_MOESM1_ESM.pdf]

nature genetics

Supplementary information

https://doi.org/10.1038/s41588-025-02496-5

Mutational scanning reveals oncogenic *CTNNB1* mutations have diverse effects on signaling

In the format provided by the authors and unedited

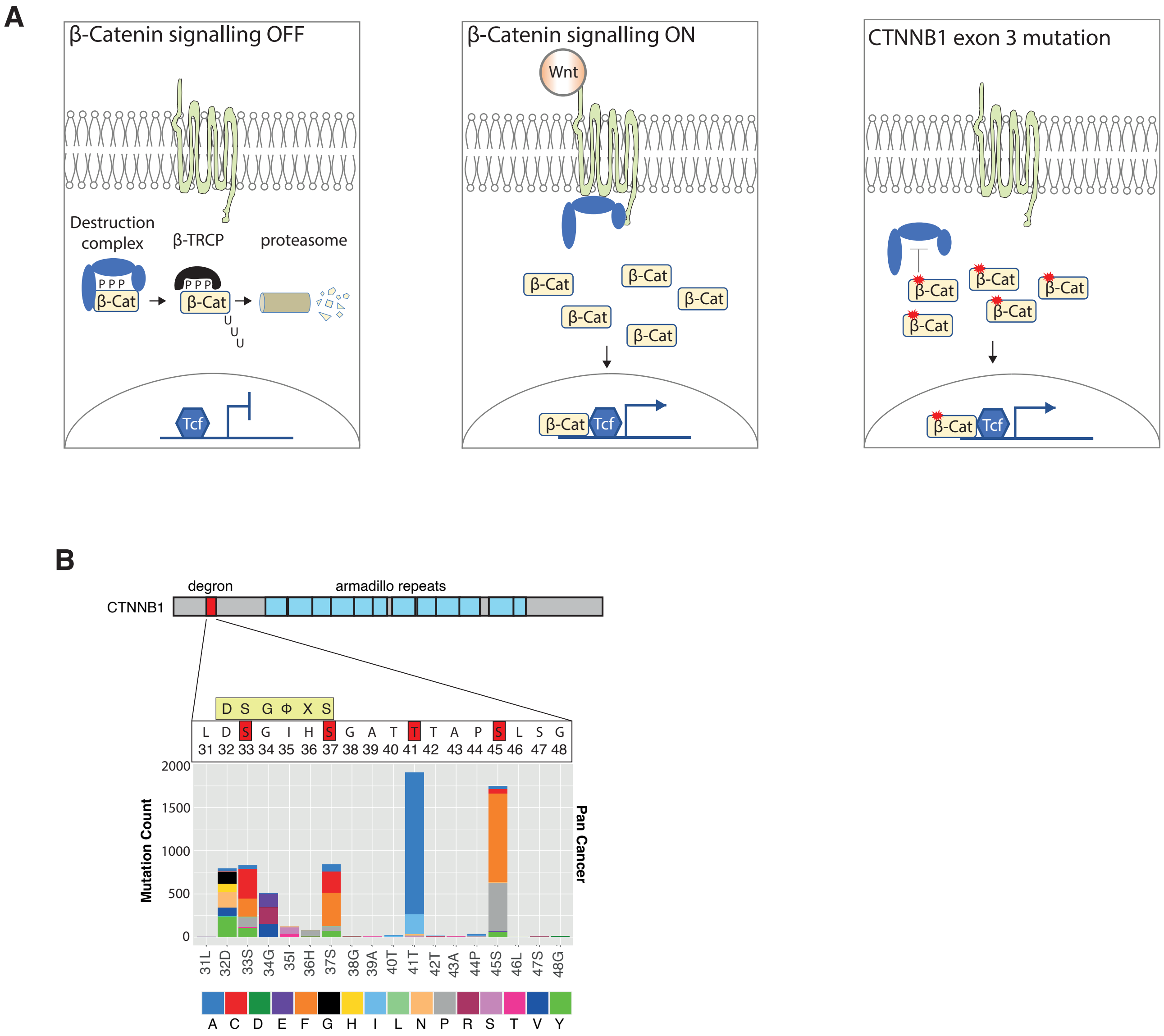

Supplementary Figure 1: Disruption of β-catenin signalling in cancer

A. Simplified schematic of canonical Wnt pathway regulation in normal development and in the presence of exon 3 degron mutations. (Left) In the absence of Wnt ligand, the destruction complex phosphorylates sites within the exon 3 degron to allow docking of the E3 ligase receptor β-TRCP, followed by ubiquitination and degradation via the proteasome. (Centre) In the presence of Wnt ligand, the destruction complex is sequestered at the cell membrane. ß-Catenin accumulates and translocates to the nucleus where it associates with TCF transcription factors to activate target genes. (Right) Exon 3 mutations prevent destruction complex activity to uncouple ß-Catenin accumulation from Wnt ligand binding, locking the pathway in the active state. **B.** Histogram showing the frequency of mutations at each amino acid position of human *CTNNB1* across all tumours present in the COSMIC database. Blue boxes represent the positions of armadillo repeat domains.

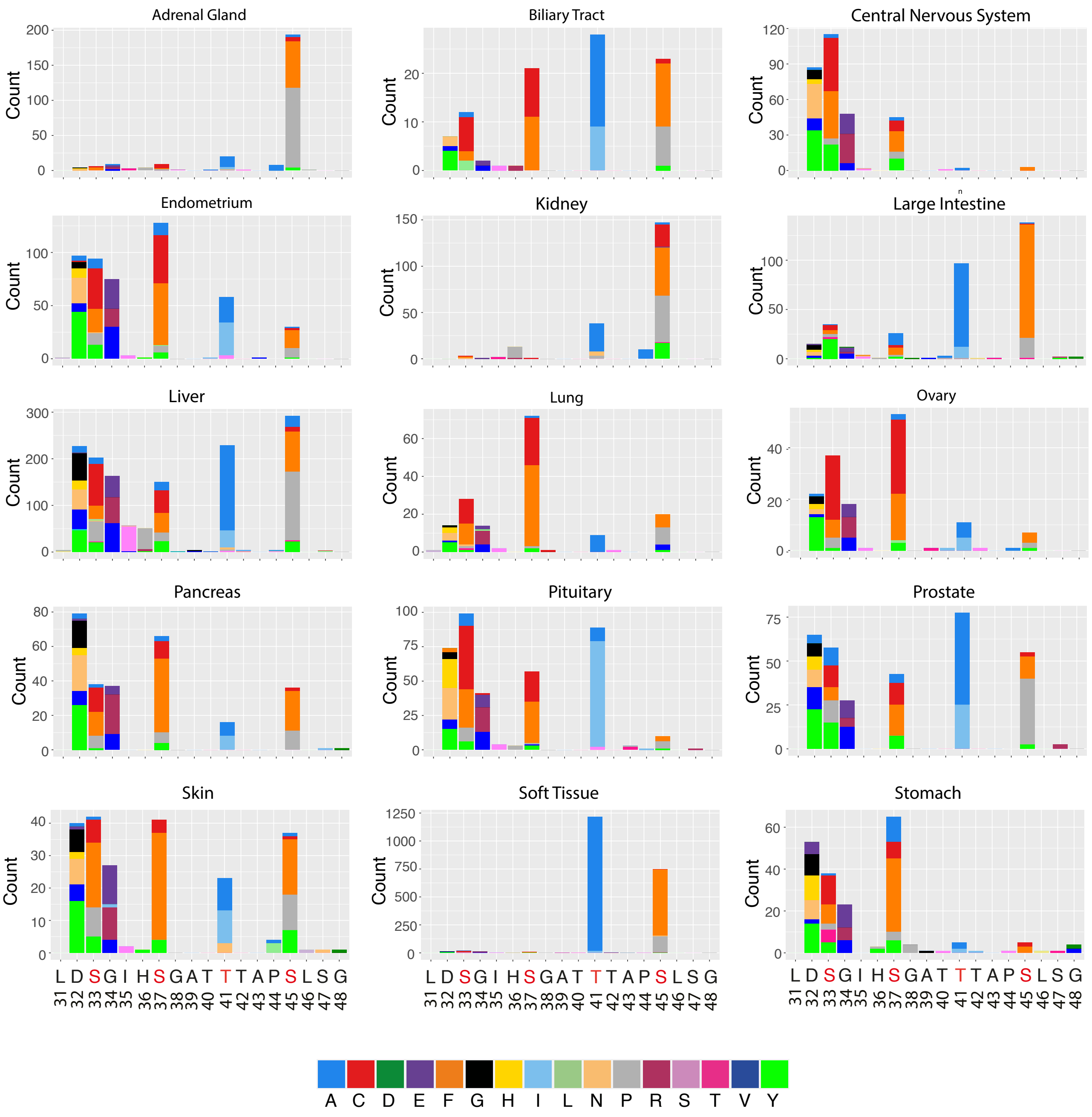

Supplementary Figure 2: Tissue-specific mutation profiles at the *CTNNB1* degron in human cancer

Histograms show distinct distributions of mutations within the β-catenin mutation hotspot for COSMIC tumours filtered by different primary tissue sites. Only tumours with >100 mutations within the exon 3 hotspot are shown. Plots showing data for Adrenal gland, Central Nervous System and Skin are the same as those shown in Figure 1C.

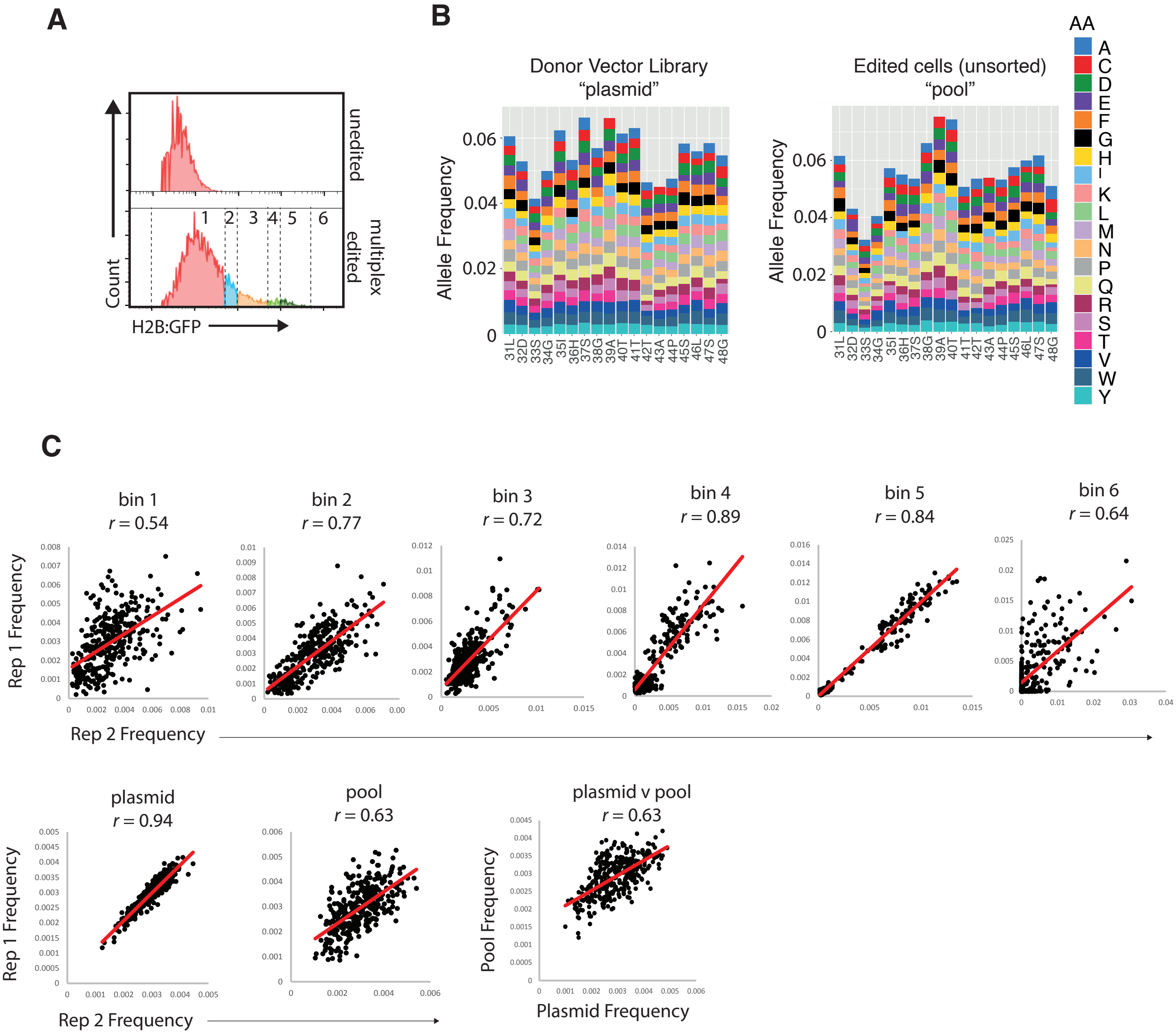

Supplementary Figure 3: Supporting data for the mutational scanning assay in Figure 1.

A. Flow cytometry histograms of cells subjected to the steps indicated in panel C, with (bottom) versus without (top) multiplex editing. **B.** The frequency of individual missense mutations in the "plasmid" library (left) used for homology-directed repair, and in the cellular "pool" (right) after editing and selection but before sorting based on GFP expression. The colour scheme used to represent different missense mutations is shown to the right. The "pool" sample was used to determine enrichment of individual mutations in each fluorescence bin (Figure 1E) during the calculation of mutational effect scores (Figure 2A). **C.** Scatter plots show correlations between experimental replicates for the fraction of sequence reads corresponding to each mutation across each bin of GFP expression, plasmid and pool control libraries.

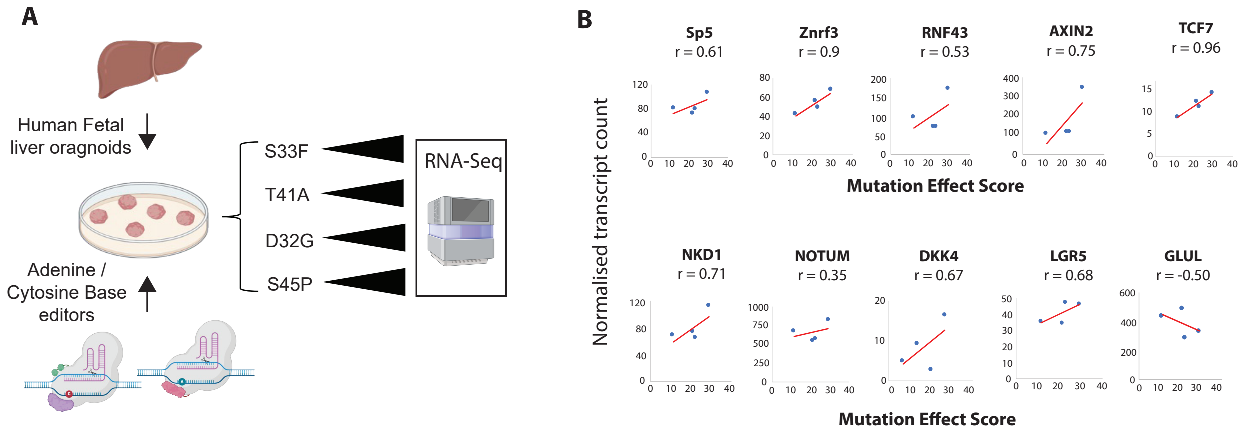

Supplementary Figure 4: β-catenin target gene expression in base edited human fetal liver organoids

A Schematic outline of the procedure followed by Geurts et al [28] to introduce homozygous exon 3 mutations using cytidine or adenine base editors. Created in BioRender. Wood, A. (2025) https://BioRender.com/errghz3 **B**. Scatter plots show correlation between mutation effect scores for the 4 mutations shown in panel A and the expression of liver β-catenin target genes. Normalised transcript counts were plotted as the mean of n = 2 biological replicate RNA-Seq experiments.

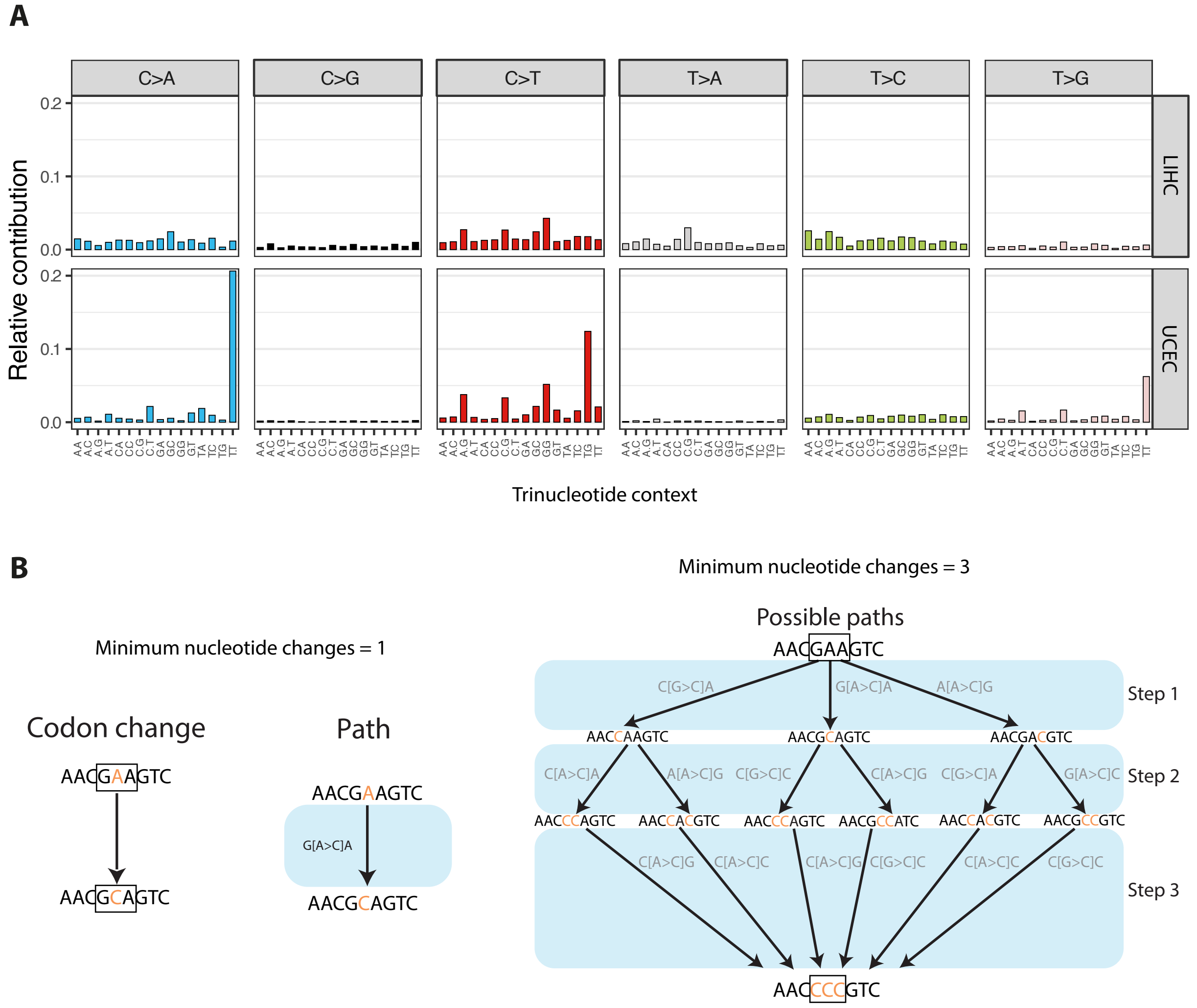

Supplementary Figure 5: Calculating Mutational Likelihood Scores for amino acid substitutions in the exon 3 hotspot

A. Histograms show the relative frequency of all possible nucleotide substitutions, calculated in all possible trinucleotide contexts (n = 96), from exome sequencing data from TCGA Hepatocellular Carcinoma (LIHC – top, n = 82) and Uterine Endometrial Carcinoma (UCEC – bottom, n = 104). Only tumours with exon 3 *CTNNB1* hotspot mutations were used. **B.** Model to illustrate the process through which nucleotide-level mutation probabilities shown in panel A are converted to amino acid-level mutational likelihood scores (MLS). Amino acid substitutions can be reached by a minimum of either 1, 2 or 3 steps. The left panel shows a simple example where only a single nucleotide change is required to reach a destination codon. Where >1 change is required (example in the right panel), the probability of alternative paths is combined. In all cases, the probability of all paths to different triplets encoding the same amino acid are integrated to generate the MLS. Further details are provided in the Methods section.

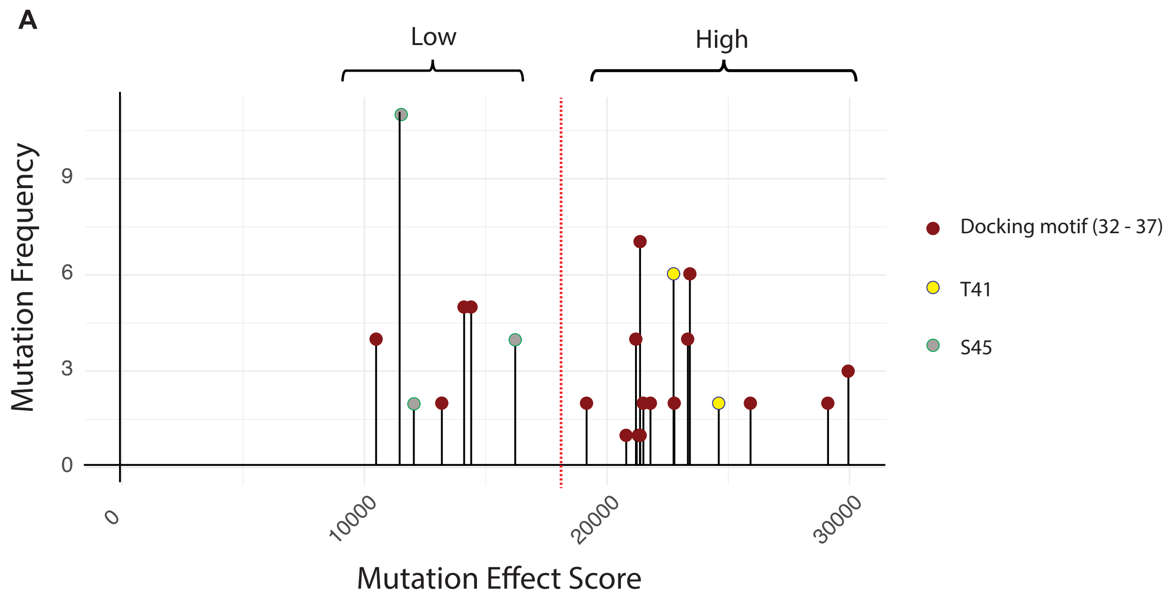

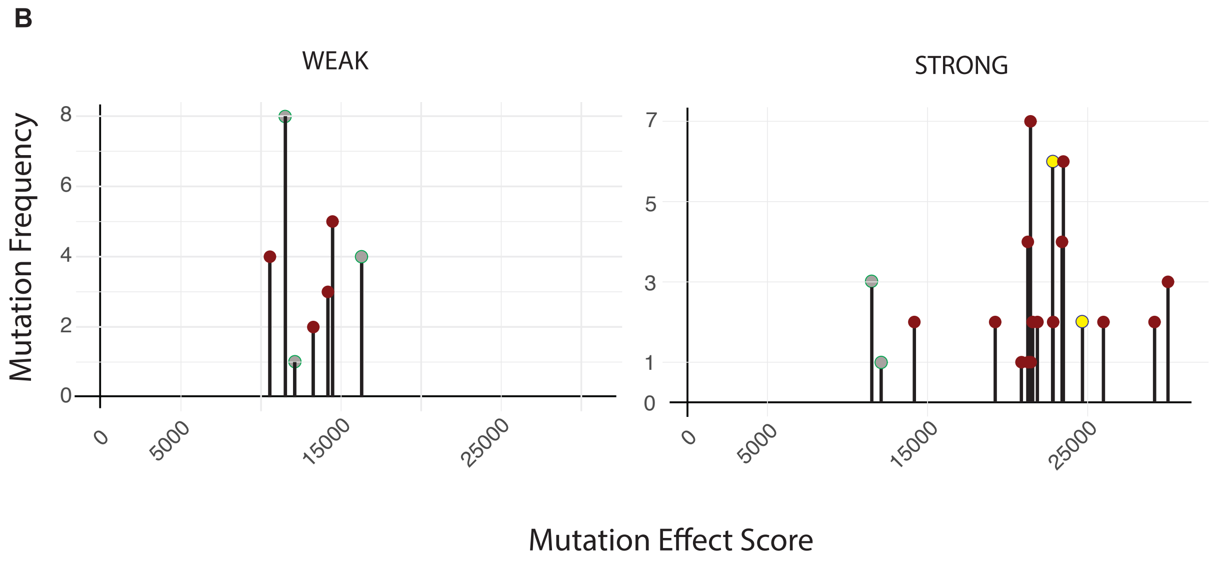

Supplementary Figure 6: Mutation effect scores for *CTNNB1* exon 3 mutations observed in Hepatocellular Carcinoma
A. Lollipop plot shows the distribution of MES values for all *CTNNB1* exon 3 mutations observed in HCC patients from the TCGA cohort. A natural gap between MES values of 16300 and 19200 was used as a threshold (red dashed line) to distinguish mutations of Low (n = 33) and High (n = 47) effect. **B.** Lollipop plots show the distribution of MES values associated with mutations assigned to the WEAK (n = 27) and STRONG (n = 53) patient groups. The STRONG group includes n = 6 mutations from the Low MES group in panel A that also harbour copy number gain spanning the mutant *CTNNB1* allele.

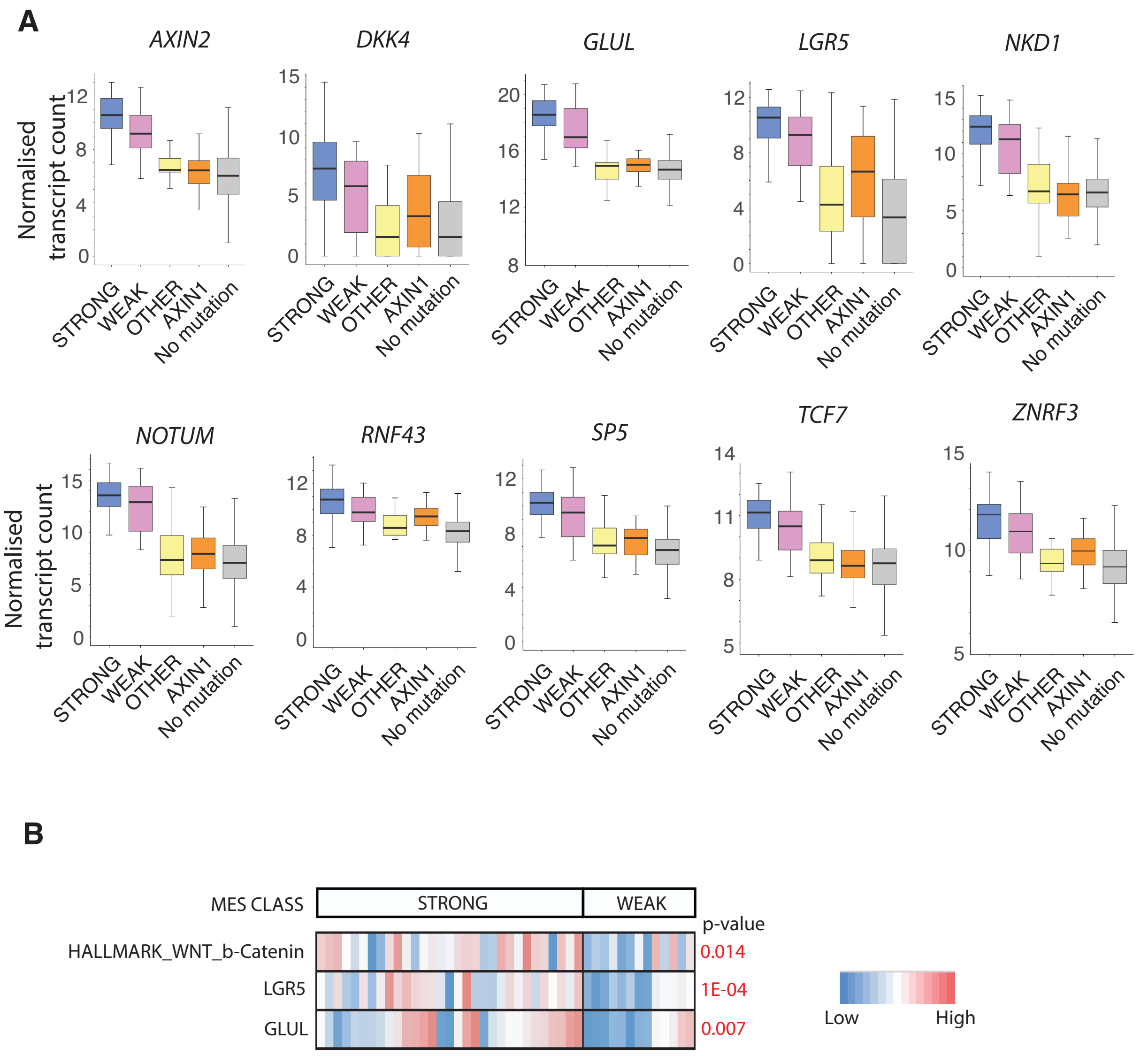

Supplementary Figure 7: *CTNNB1* mutation effect scores predict signalling activation in Hepatocellular Carcinoma

A. Expression of 10 canonical β-catenin target genes in TCGA Hepatocellular Carcinoma samples stratified as indicated in Figure 5A. Median expression values from each gene in each group are shown in Figure 5C. Horizontal lines show the median value, boxes show the upper and lower quartiles and whiskers show the range.

B. Significant association of *CTNNB1* mutation strength and signalling activation in a second HCC cohort [14]. Heatmap representation of normalised transcript abundance values for 42 genes known to be upregulated by the accumulation of β-catenin (Hallmark_wnt_b-Catenin gene set, MSigDB), and two HCC targets (LGR5, GLUL). Only patient samples with exon 3 mutations are shown. p-values indicate 1-tailed t-tests.

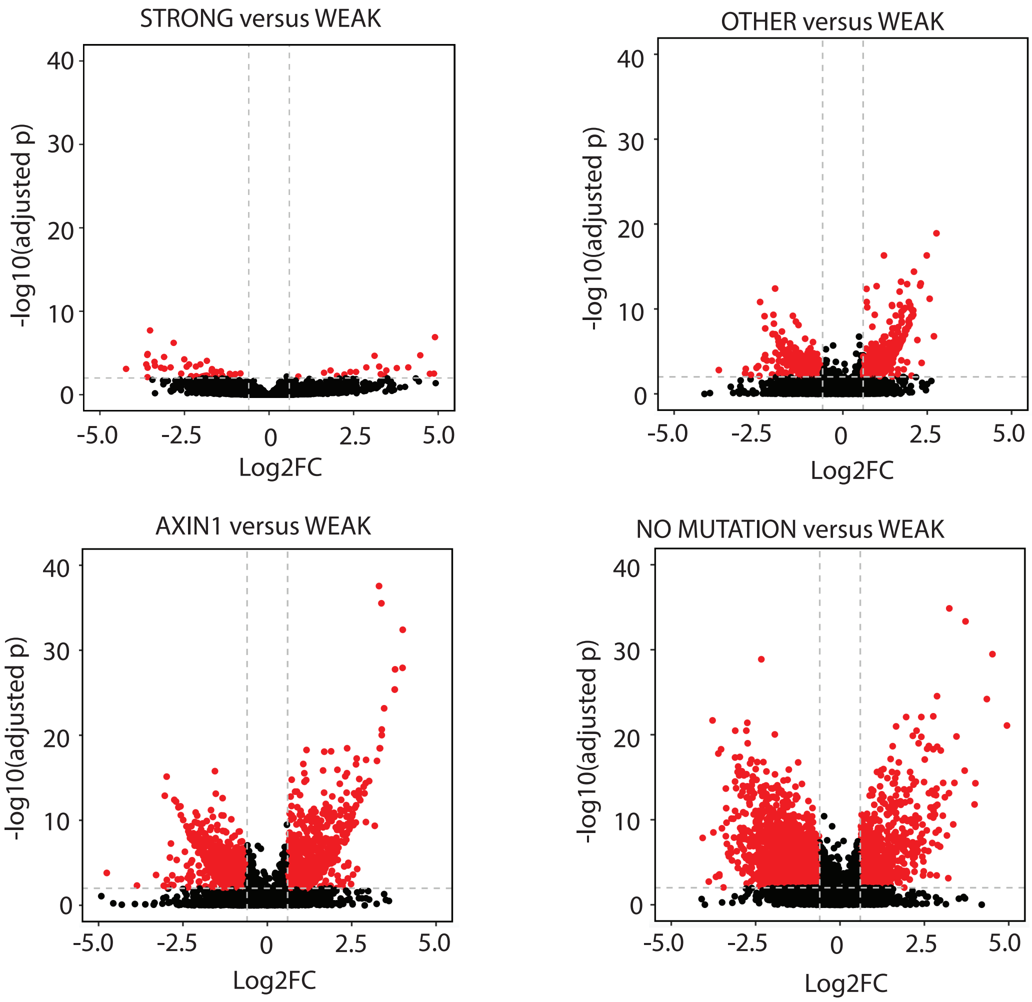

Supplementary Figure 8: Transcriptomic differences between HCC samples grouped by β-catenin pathway mutation status
Volcano plots show the Log2 fold change and -Log10 adjusted p-values for differentially expressed genes in transcriptome comparisons between tumours with WEAK mutations in the *CTNNB1* hotspot region and other classes shown in Figure 5A. Dotted lines indicate significance thresholds (Log2FC greater than 0.6, adjusted p value < 0.01), and points coloured red denote genes which pass these thresholds. The values for individual genes are shown in Supplementary Table 5, and Gene Ontology term enrichments in Supplementary Table 6.

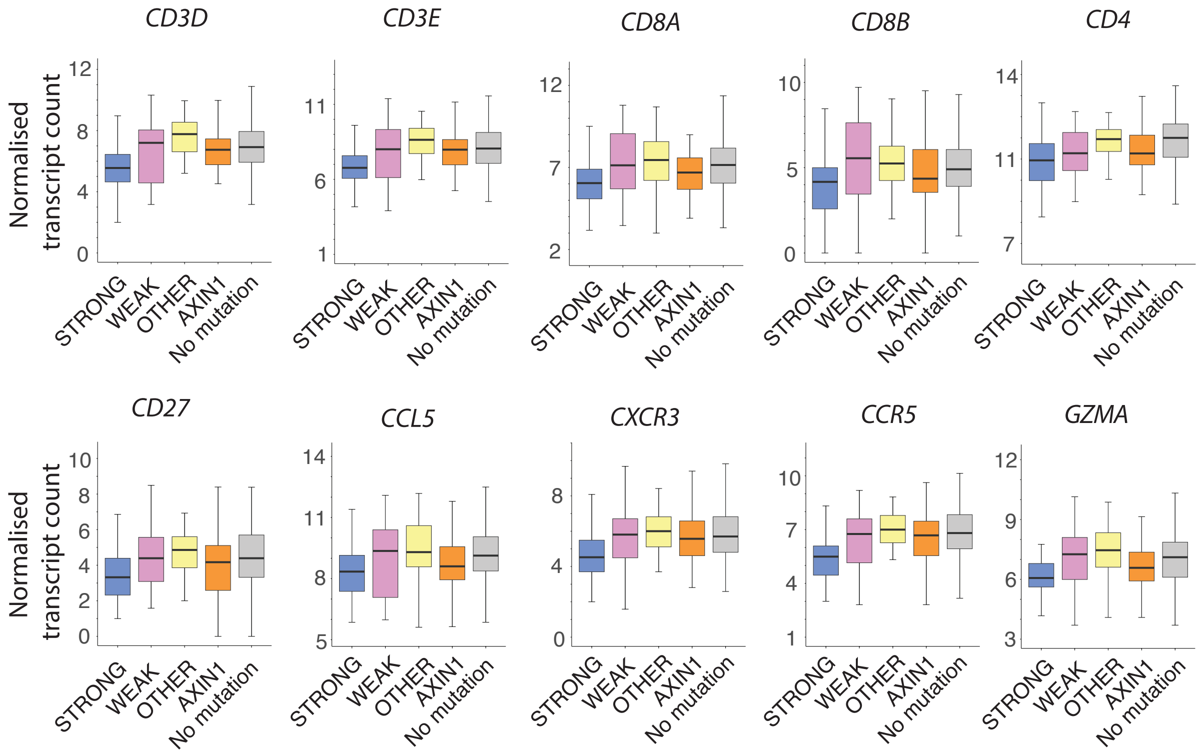

Supplementary Figure 9: *CTNNB1* mutation effect scores predict T cell gene expression in Hepatocellular Carcinoma

Expression of 10 T cell signature genes in TCGA Hepatocellular Carcinoma samples stratified as indicated in Figure 5B. Horizontal lines show the median value, boxes show the upper and lower quartiles and whiskers show the range.
